# A bronchofiberoscopy-associated outbreak of multidrug-resistant *Acinetobacter baumannii* in an intensive care unit in Beijing, China

**DOI:** 10.1186/1471-2334-12-335

**Published:** 2012-12-03

**Authors:** Yukun Xia, CuiLing Lu, Jingya Zhao, Gaige Han, Yong Chen, Fang Wang, Bin Yi, Guoqin Jiang, Xiaohua Hu, Xianfeng Du, Zheng Wang, Hong Lei, Xuelin Han, Li Han

**Affiliations:** 1 Department for Hospital Infection Control & Research, Institute of Disease Control & Prevention of People’s Liberation Army, Academy of Military Medical Sciences, Fengtai Dong Street 20, Beijing, China; 2 309 hospital of the Chinese People’s Liberation Army, Beijing, China; 3 Department of Microbiology, School of Basic Medical Sciences, Central South University, Changsha, Hunan, China; 4 General Hospital of Chinese People’s Liberation Army, Beijing, China

**Keywords:** Outbreak, Bronchofiberscopy, Multidrug-resistant *Acinetobacter baumannii*

## Abstract

**Background:**

Bronchofiberscopy, a widely used procedure for the diagnosis of various pulmonary diseases within intensive care units, has a history of association with nosocomial infections. Between September and November 2009, an outbreak caused by multidrug-resistant *Acinetobacter baumannii* (MDR-Ab) was observed in the intensive care unit of a tertiary care hospital in Beijing, China. This study is aimed to describe the course and control of this outbreak and investigate the related risk factors.

**Methods:**

Clinical and environmental sampling, genotyping with repetitive extragenic palindromic polymerase chain reaction (REP-PCR), and case–control risk factor analysis were performed in the current study.

**Results:**

During the epidemic period, 12 patients were infected or colonized with MDR-Ab. Sixteen (72.7%) of twenty-two MDR-Ab isolates from the 12 patients and 22 (84.6%) of 26 MDR-Ab isolates from the bronchofiberscope and the healthcare-associated environment were clustered significantly into a major clone (outbreak MDR-Ab strain) by REP-PCR typing. Seven patients carrying the outbreak MDR-Ab strain were defined as the cases. Six of the seven cases (83%) received bronchofiberscopy versus four of the 19 controls (21%) (odds ratio, 22.5; 95% confidence interval, 2.07–244.84; P = 0.005). Several potential administrative and technical problems existed in bronchofiberscope reprocessing.

**Conclusions:**

Bronchofiberscopy was associated with this MDR-Ab outbreak. Infection control precautions including appropriate bronchofiberscope reprocessing and environmental decontamination should be strengthened.

## Background

Multidrug-resistant *Acinetobacter baumannii* (MDR-Ab), one of the most important healthcare-associated pathogens worldwide, causes infections such as hospital-acquired pneumonia, wound infections, meningitis, endocarditis, and bloodstream infections (BSIs) due to its prolonged environmental survival and extensive resistance to many of the currently available antibiotics, including cephalosporins, aminoglycosides, quinolones, and carbapenems [1]. Nosocomial MDR-Ab infection most commonly occurs in intensive care units (ICUs), although epidemic strains have also been isolated in other hospital departments [2,3]. MDR-Ab outbreaks in ICUs have been reported to be associated with various types of devices and medical procedures used in patient management [4,5]. Most relevant reports have referred to medical devices and procedures used for respiratory systems, such as mechanical ventilators, laryngoscope blades, and tracheostomy equipment [6-8]. In addition, long hospital or ICU stays, exposure to infected or colonized patients in neighboring hospital environments, infection with a critical illness, and the receipt of broad-spectrum antimicrobial agents are very important factors of MDR-Ab transmission throughout institutions during outbreaks [9-12]. MDR-Ab–induced BSI outbreaks have also been reported, and the clinical manifestations of MDR-Ab BSIs may range from transient bacteremia to septic shock and fulminating disease accompanied by an overall mortality (case-fatality ratio) as high as 46% [13-15].

Bronchofiberscopy, the visual examination of the tracheobronchial tree using a fiberoptic bronchofiberscope, is currently an indispensable tool within ICUs. Several nosocomial infections caused by *Pseudomonas aeruginosa*, *Serratia marcescens, Mycobacteria*, and others have been reported to be associated with bronchofiberscopy and the reprocessing of bronchofiberscopes, such as lacking cleaning and disinfection procedures, [16] problems related to bronchoscopy suites, [17-19] and device defects (e.g., loose biopsy port caps, damage due to prolonged physical use) [20-22]. However, to date, no report has detailed the involvement of bronchofiberscopy in MDR-Ab outbreaks. In September 2009, the Department for Hospital Infection Control & Research, Institute for Disease Control & Prevention of PLA, China received a report from an ICU in a 1,200-bed hospital in Beijing that a cluster of five patients had healthcare-associated MDR-Ab–induced BSIs. Therefore, an outbreak investigation was conducted between September 2009 and January 2010 to describe its course and control and find its related risk factors. This study is the first to describe a nosocomial MDR-Ab outbreak related to bronchofiberscopy.

## Methods

### Ethics statement

This study was approved by the institutional ethics committees of the Academy of Military Medical Sciences and 309 Hospital of the Chinese People’s Liberation Army, Beijing, China. Written informed consent was obtained from all participants before the study.

### Setting

The outbreak occurred in an ICU ward comprising a large open bedroom with ten beds, a buffer room, treatment room, and equipment room. Every bed was equipped with an alcohol-based hand rub. Fifteen doctors and thirty-one nurses worked in this ICU and approximately 12 nurses were on duty every day. There was only one bronchofiberscope in the ICU and bronchofiberscopy was performed once or twice each day for diverse examination and treatment indications such as corpus alienum removal, secretion clearance, tracheal intubations, and bronchoalveolar lavage. After each procedure, the bronchofiberscope was reprocessed by the professional staff in the Center for Cleaning and Disinfection of the hospital according to the Chinese guidelines for endoscopy cleaning and disinfection [23]. The standard procedure for reprocessing a bronchofiberscope includes the following steps: pre-cleaning, cleaning with an enzymatic detergent, rinsing, disinfecting, final rinsing, drying, and storing. However, when a bronchofiberscope was used emergently and frequently, it was reprocessed directly and manually by a doctor in the ICU after each use. Neither a doctor nor a nurse was specifically appointed to reprocess the bronchofiberscope and no automatic reprocessing machine was used.

### Epidemiological investigation

During the epidemic period from 5^th^ August 2009 to 30^th^ November 2009, 153 patients were admitted to the ICU. The period from 1^st^ January 2009 to 4^th^ August 2009 was considered the pre-epidemic period. Medical records including paper and electronic charts were reviewed. Microbiological records were carefully analyzed to screen the cases and define the baseline MDR-Ab rate before the outbreak. Any patient who had at least one clinical or screening sample that was positive for a MDR-Ab who had the corresponding clinical symptoms (e.g., pneumonia, bacteremia, peritonitis) detected at least 48 h after ICU admission was noted. Multidrug resistance was defined as resistance to ≥3 of the following classes of antibiotics: penicillins, cephalosporins, aminoglycosides, fluoroquinolones, and carbapenems [24]. Environmental sampling was performed on 15^th^, 21^st^, and 28^th^ October 2009. Samples were taken from the hands and nasal cavities of the ICU staff as well as multiple surfaces within the ICU environment including: bed sheets, bedrails, and bedside tables associated with cases and controls; healthcare workers’ clothes, computer keyboards, and calculators; and the surfaces of invigilators, ventilators, hemofiltration machines, bronchofiberscopes, electrocardiography machines, ultrasound machines, and laryngeal endoscopes.

### Case–control study

The case–control study was conducted to investigate this outbreak’s risk factors. Blood, urine, sputum, wound, bile, and catheter cultures were processed. A case was defined as a patient with at least one isolate identified as the outbreak MDR-Ab strain in clinical culture (outbreak strain carrier) at least 48 h after ICU admission during the period of 1^st^ September 2009 to 31^th^ October 2009. A control was defined as a patient who stayed ≥ 48 h in the ICU during the same period without the identification of an outbreak strain in any clinical culture [25]. Patients who stayed in the ICU < 48 h, carried strains other than the outbreak strain as determined by repetitive extragenic palindromic polymerase chain reaction (REP-PCR), or harbored MDR-Ab before the ICU admission were excluded from the study. The ratio of controls to cases was 2.7:1. At least one clinical urine, sputum, wound, or blood culture was processed for each control during the study period.

For the case–control study, the presence of primary diseases or medication history including septic shock, multiple organ failure, pulmonary diseases, renal diseases, and surgical operation was determined at the time of ICU admission. For invasive and other bedside procedures such as blood transfusion, mechanical ventilation, bedside diagnostic ultrasonography, bedside chest X-ray, bronchofiberscopy, electrocardiography, venipuncture, gastric lavage, urinary catheterization, and hemodialysis, the presence of a central line, and antibiotic use, the observation period lasted from ICU admission to outbreak strain detection for the cases and from ICU admission to patient discharge for the controls. ICU stay and hospital stay were defined as the length of stay until outbreak strain detection for the cases, whereas they were defined as the length of stay until patient discharge in the controls.

### Microbiological methods

Swabs from environmental samples and healthcare workers were inoculated on blood plates. Colonies resembling *Acinetobacter* spp. were then isolated and transferred onto a China-Blue lactose agar plate (Luqiao, Beijing, China). The *A. baumannii* isolates were further identified according to their morphological and growth characteristics using the oxidase, triple sugar iron, and citrate tests. The clinical samples were also inoculated in blood agar and the isolates were identified using the automated Microscan-Walkaway Microbiology Identification System (Becton Dickinson, Sparks, MD, USA). Antimicrobial susceptibility was determined using the disk diffusion method and interpreted according to Clinical Laboratory Standards Institute guidelines. DNA extractions of the isolates were typed by REP-PCR using REP1 (5^′^-IIIICGICGICATCIGGC-3^′^) and REP2 (5^′^-ICGICTTATCIGGCCTAC-3^′^) primer sequences [26]. Characteristic DNA patterns were analyzed using BioNumerics software (version 3.0; Applied Math, Sint-Martens-Latem, Belgium) to determine the distance matrices and the unweighted pair group method with arithmetic mean method to create a dendrogram.

### Intervention

Three major interventions were implemented on 21^st^ October 2009. First, the bronchofiberscope reprocessing by doctors within the ICU was stopped and the bronchofiberscope was sent to the Center for Cleaning and Disinfection of the hospital for professional reprocessing. Another one or two bronchofiberscopes were prepared for use in emergent situations in the ICU. Second, surveillance culturing for MDR microorganisms from the bronchofiberscope was performed regularly after every reprocessing round. Third, the ICU environmental surfaces were cleaned thoroughly and disinfected with a solution containing electrolyzed acid water according to the manufacturer’s instructions. Fourth, education and training were enhanced for endoscopy reprocessing and general infection control procedures in this ICU. In addition, to investigate the effect of hand hygiene on this outbreak, healthcare workers’ hand hygiene compliance was observed as described previously [27]. Briefly, infection control professionals recorded opportunities for hand hygiene during 1-h observation periods distributed randomly between 8:00 a.m. and 5:00 p.m. every day during the investigation. Hand hygiene compliance prior to the outbreak was calculated by review of the video documentation in the ICU. Healthcare workers’ hand hygiene compliance rose from 30% to 90% after the intervention.

### Statistical analysis

Statistical analyses were performed using SAS software (version 8.1; SAS Institute Inc., Cary, NC, USA). The Chi-square test was used to compare the differences of MDR-Ab incidences between the pre-epidemic and epidemic periods. In the case–control study, cases and controls were compared using the Mann–Whitney *U*-test or Student’s *t*-test for continuous variables and using Fisher’s exact test for categorical variables. Odds ratios (ORs) and 95% confidence intervals (CIs) were calculated for binomial variables. Two-sided P values < 0.05 were considered statistically significant. Multivariate logistic regression analysis was not applied due to the small sample size.

## Results

### Epidemiological investigation

**Figure 1:**
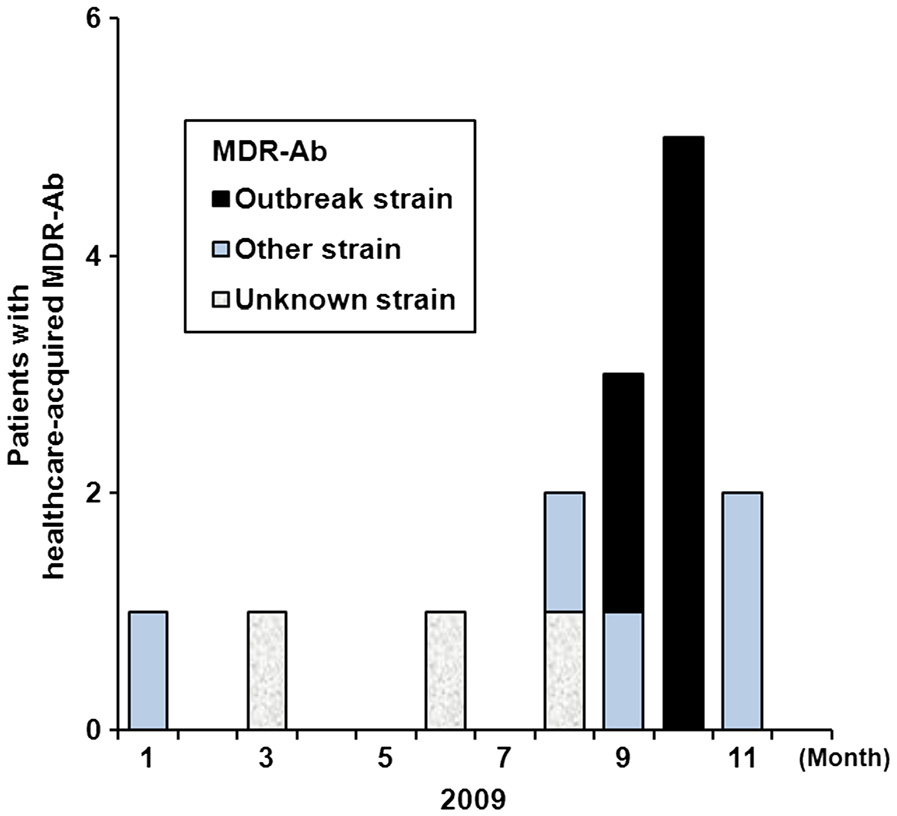
**Epidemical curve showing the rate of healthcare-associated multidrug-resistant *Acinetobacter baumannii *(MDR-Ab) in the ICU in 2009.** A marked increase in the number of cases was noted during the epidemic period. The pattern indicates various strains of MDR-Ab as defined by REP-PCR. After the outbreak was halted in late October, the number of cases decreased. Two patients still acquired MDR-Ab in November; however, the isolates from these two patients were unrelated to the outbreak strain. In December and the next January, no healthcare-associated MDR-Ab infections were detected.

**Figure 2:**
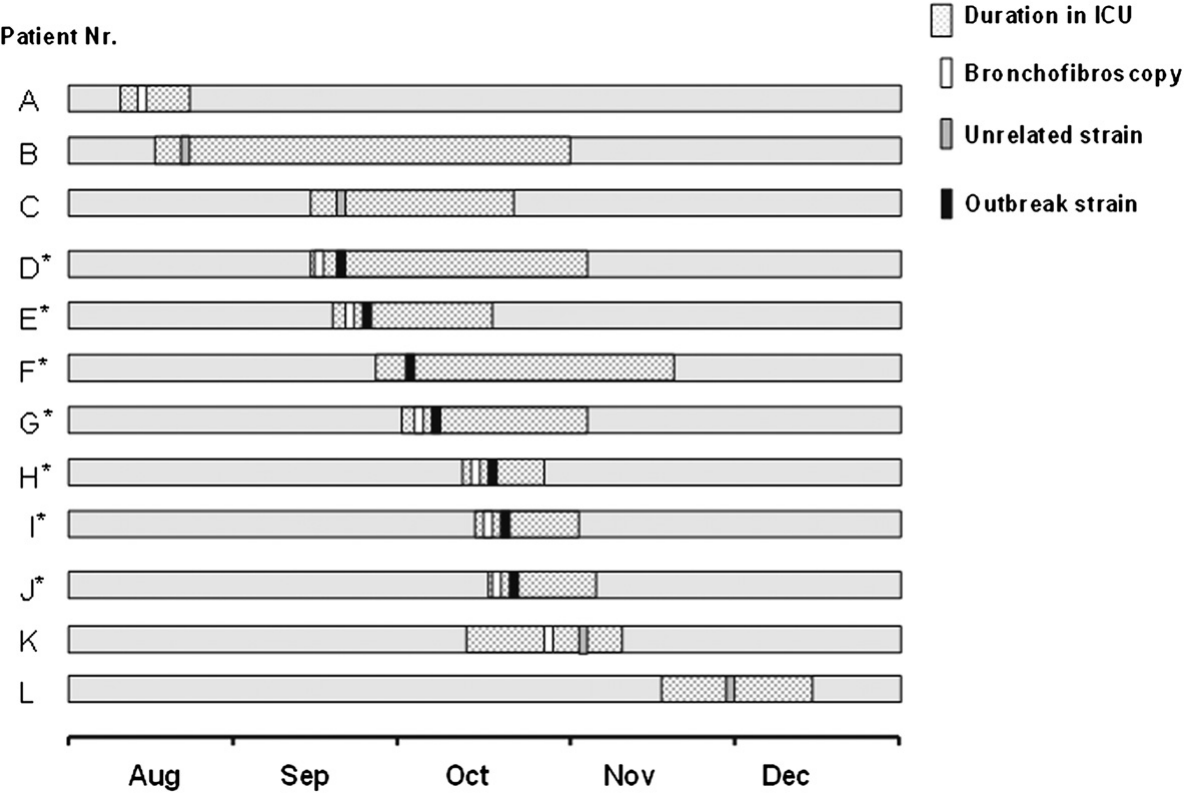
**Timeline of 12 patients with healthcare-associated multidrug-resistant *Acinetobacter baumannii *(MDR-Ab) during the epidemic period.** This timeline depicts the 12 patients who were identified as MDR-Ab carriers from August to November 2009. Patient’s duration in the intensive care unit, exposure to bronchofiberscopy, and the positive culture of unrelated strains or outbreak strain are indicated in the figure. Asterisks indicate cases.

Three patients acquired MDR-Ab infection or colonization (MDR-Ab carriers) in the ICU during the pre-epidemic period. A total of 12 patients (seven males and five females ages 39–97) were identified as MDR-Ab carriers at least 48 h after ICU admission from 5^th^ August to 30^th^ November 2009 (Figure 1, Figure 2). Compared to the pre-epidemic period, the increase of MDR-Ab incidence during the epidemic period was statistically significant (χ^2^ = 13.82, P < 0.001). Two MDR-Ab carriers who were admitted into the ICU before 20^th^ September 2009 were identified by medical record review. Five MDR-Ab carriers were identified in the 18 days between 21^st^ September and 8^th^ October 2009, while three other carriers were found in the four days between 19^th^ October and 22^nd^ October 2009. Thereafter, only two MDR-Ab carriers were identified on 2^nd^ November and 30^th^ November, and no other MDR-Ab carriers were detected up to the end of the study in January 2010.

**Figure 3:**
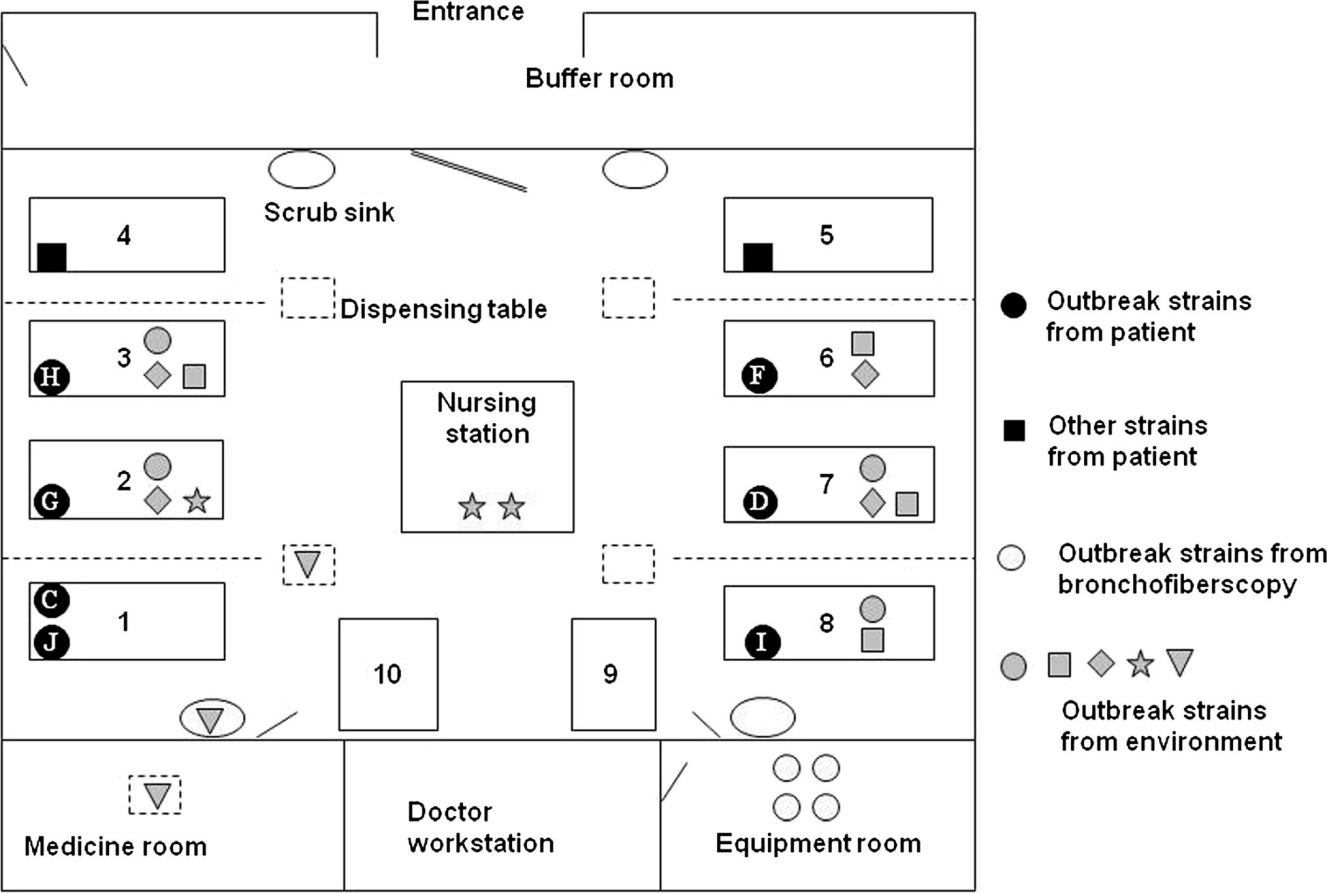
**Schematic map of the intensive care unit (ICU) and the distribution of the outbreak multidrug-resistant *Acinetobacter baumannii *(MDR-Ab) strain and the other MDR-Ab strains identified in the outbreak.** Numbers 1–10 indicate the bed number (rectangles) in the ICU. The black dots represent the outbreak strain; the white letters inside them are the patient numbers. The gray shapes represent the isolates from the environment identified as outbreak strain (●, isolates from bedrails; ■, isolates from bed sheets; ♦ isolates from invigilator or blood filtering machine keyboards; ★, isolates from nurses’ notebook, desk, and calculator; ▼, isolates from dispensing table, scrub sink, and the medical treatment room). The ellipsis indicates the scrub sink. The dashed lines indicate physical barriers (drapes). There was a two-meter distance between adjacent beds.

The patients had a variety of underlying conditions, including septic shock, organ transplantation, malignant tumor, respiratory failure, acute pancreatitis, chronic obstructive pulmonary disease, multiple organ dysfunction syndrome, and coronary heart disease. Seven of the 12 patients were identified when they were staying in beds 1, 2, 3, 6, 7, and 8 (Figure 3). The average interval between ICU admission and MDR-Ab identification was 6.3 ± 3.8 days. Eight of the 12 patients had received bronchofiberscopy and five had BSIs. Six patients (50%) died in the ICU and three patients’ deaths (B, D, E) were possibly related to MDR-Ab infection (Table 1).

**Table 1:** **Clinical characteristics of multidrug-resistant *Acinetobacter baumannii *(MDR-Ab) carriers from 5^th^ August to 30^th^ November 2009 in the intensive care unit**

**Patient**	**MDR-Ab culture site**	**Bronchofiberscopy**	**Patient outcome**	**MDR-Ab strain**
A	Sputum	Yes	Survived	NA
B	Ascites, sputum	No	Died	G
C	Sputum, blood	No	Survived	C
D	Blood, sputum, catheter	Yes	Died	A
E	Blood, sputum, pleural fluid	Yes	Died	A
F	Bile, catheter, sputum	No	Survived	A
G	Blood, sputum, catheter	Yes	Died	A
H	Blood, sputum, catheter	Yes	Died	A
I	Sputum	Yes	Survived	A
J	Blood, sputum, wound	Yes	Survived	A
K	Sputum	Yes	Died	B
L	Sputum	No	Survived	D

*MDR-Ab* multidrug-resistant *Acinetobacter baumannii*; *NA* isolate not available for analysis.

**Figure 4:**
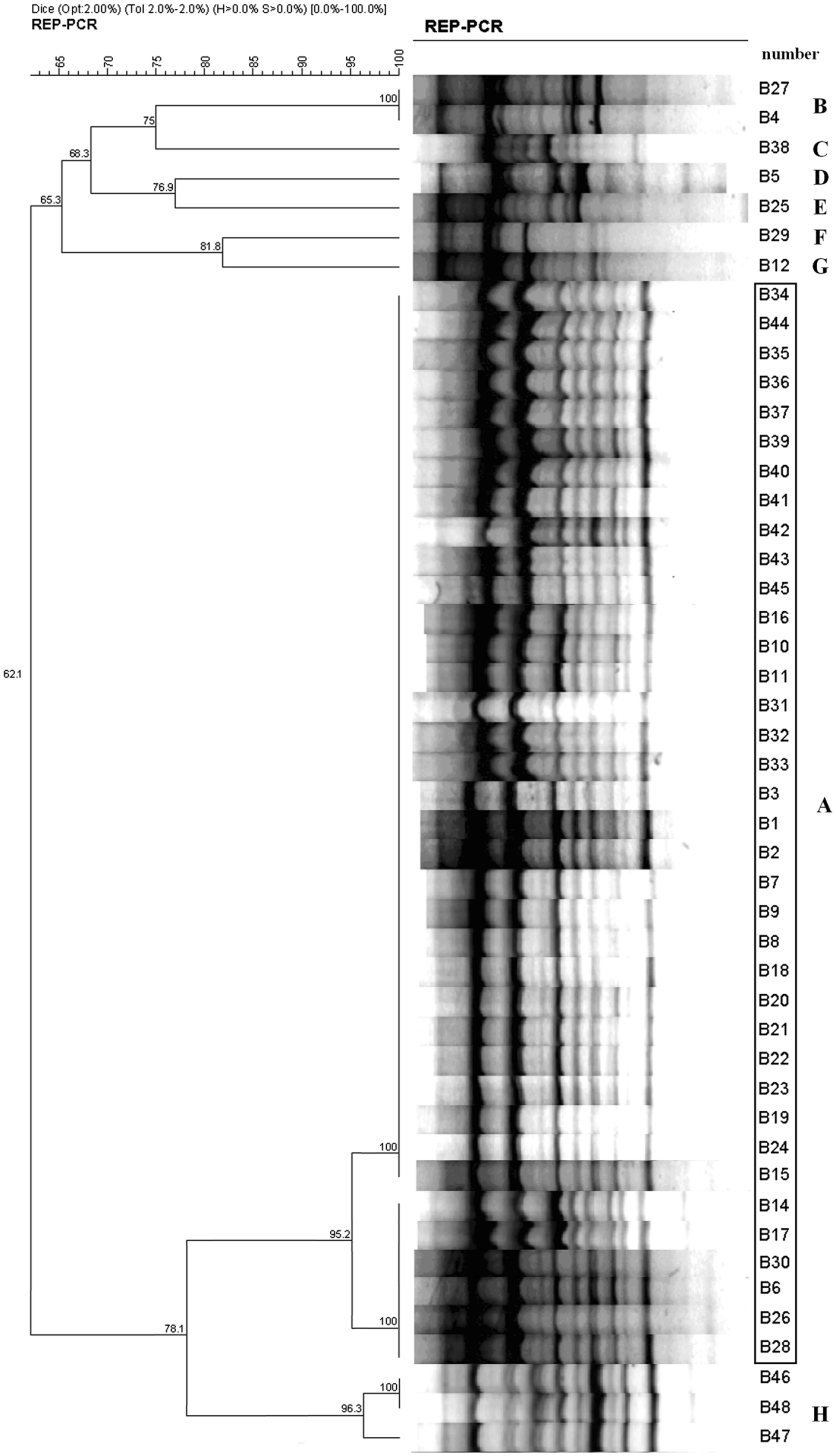
**Cluster analysis of multidrug-resistant *Acinetobacter baumannii *(MDR-Ab) isolates by repetitive extragenic palindromic polymerase chain reaction (REP-PCR).** Forty-eight MDR-Ab isolates were classified into eight genotypes according to 90% similarity by REP-PCR. Among these, 38 isolates inside the long panel demonstrate type A, the major type. The other isolates were not identical and corresponded to other types. A percent genetic similarity scale is shown above the dendrogram. Band position tolerance and optimization were each set at 2.0%.

A total of 22 MDR-Ab isolates were available from seven patients who underwent bronchofiberscopy and from four patients who did not undergo bronchofiberscopy. All of the MDR-Ab isolates were completely resistant to cefuroxime, cefotaxime, imipenem, and levofloxacin but were susceptible to colistin. REP-PCR analysis revealed that 16 MDR-Ab isolates from six patients who received bronchofiberscopy shared an identical outbreak strain of MDR-Ab (genotype A) that differed from the isolates (genotypes C, D, G) obtained from three of the patients who did not undergo bronchofiberscopy (Figure 4, Table 1). Four patients who received bronchofiberscopy during the epidemic period did not test positive for MDR-Ab.

Environmental sampling showed that 26 MDR-Ab isolates were identified from 78 environmental samples on 15^th^ October 2009, whereas no MDR-Ab isolates were detected in the later environmental samples on 21^th^ and 28^th^ October 2009. A total of 22 of 26 MDR-Ab isolates (84.6%) were identical to the outbreak strain. Of these 22 isolates, four were recovered from the biopsy forceps and the bronchofiberscope tip that were used to treat a patient carrying the outbreak strain and from the bronchofiberscope surface after reprocessing within the ICU. Of the 22 isolates, 13 were recovered from the bed sheets, bedrails, dispensing table, and invigilator or blood filtering machine keyboard of beds 1–3 and 6–8, findings that were in line with the distribution of the outbreak strains within the patients. Five of 22 isolates were detected in the scrub sink, the medical treatment room, and the nurses’ notebook, desk, and calculator (Figure 3). However, no MDR-Ab isolates were detected from the healthcare workers’ hand or nasal cavity samples.

Further investigation disclosed several potential administrative and technical problems in the bronchofiberscope reprocessing protocol. First, from the end of July 2009, bronchofiberscope was frequently reprocessed in the ICU by doctors after emergent patient examinations and treatments. Second, the bronchofiberscope reprocessing procedure was not strictly in accordance with the Chinese guidelines for endoscopy cleaning and disinfection [23]. For instance, the pre-cleaning time was not adequate and the specific enzyme-containing detergent was seldom used. In addition, the patients who received bronchofiberscopy were seldom covered during emergent treatment, and the potentially contaminated environmental surface was not disinfected immediately and thoroughly after the bronchofiberscopy procedure was performed.

### Case–control study

The cases and controls were similar with respect to age, sex, and hospital length of stay (Table 2). Univariate analysis confirmed that bronchofiberscopy was a significant risk factor for MDR-Ab acquisition. Six of the seven cases (83%) were treated with bronchofiberscopy versus four of the 19 controls (21%) (OR, 22.5; 95% CI, 2.07–244.84; P = 0.005). The cases had higher rates of septic shock and renal disease than did the controls (P < 0.05), indicating that the cases had more serious underlying diseases. Five of seven cases compared to only one control received carbapenem (OR, 45; 95% CI, 3.35–603.99; P = 0.002). Other significant factors included ICU stay length, bedside diagnostic ultrasonography, and bedside chest X-ray (P < 0.05). None of the other variables tested, including mechanical ventilation, the presence of a central line, pulmonary diseases, blood transfusion, and fluoroquinolone administration, differed significantly between cases and controls (Table 2).

**Table 2:** **Comparison of selected risk factors for healthcare-associated infection or colonization with multidrug-resistant *Acinetobacter baumannii *in the intensive care unit from 1^st^ September to 31^st^ October 2009**

**Risk factors**	**No. (%)**		**Odds ratio (95% CI)**	**P value**
	**Cases**	**Controls**		
	**(n = 7)**	**(n = 19)**		
Age, y (mean (SD))	67.1 (22.9)	67.2 (16.9)	-	0.99
Male	5 (71.4)	12 (63.2)	1.46 (0.22–9.62)	1
Hospital stay, days [median (IQR)]	7 (4–61)	9 (3–47)	-	1
ICU stay, days [median (IQR)]	6 (4–8)	3 (2–6)	-	0.001
Blood transfusion	5 (71.4)	13 (68.4)	1.15 (0.17–7.74)	1
Mechanical ventilation	6 (85.7)	16 (84.2)	1.13 (0.10–13.04)	1
Bedside diagnostic ultrasonography	6 (85.7)	5 (41.7)	16.8 (1.60–176.23)	0.02
Bedside chest X-ray	7 (100.0)	4 (36.4)	-	<.001
Bronchofiberscopy	6 (85.7)	4 (21.1)	22.50 (2.07–244.84)	0.005
Electrocardiography	1 (14.3)	3 (15.8)	0.89 (0.08–10.30)	1
Venipuncture	7 (100.0)	12 (63.2)	-	0.13
Gastric lavage	7 (100.0)	12 (63.2)	-	0.13
Urinary catheterization	6 (85.7)	19 (100.0)	-	0.27
Hemodialysis	3 (42.9)	2 (10.5)	6.38 (0.78–51.78)	0.10
Presence of central line	2 (28.6)	2 (10.5)	3.40 (0.38–30.66)	0.29
Surgical operation	3 (42.9)	4 (36.4)	2.81 (0.44–18.06)	0.34
Septic shock	4 (57.1)	1 (5.3)	24.00 (1.95–295.06)	0.01
Multiple organ failure	3 (42.9)	1 (5.3)	13.50 (1.10–165.89)	0.05
Pulmonary diseases	6 (85.7)	8 (42.1)	8.25 (0.82–82.67)	0.08
Renal diseases	5 (71.4)	3 (15.8)	13.33 (1.71–103.75)	0.01
Fluoroquinolone administration	2 (28.6)	3 (15.8)	2.13 (0.27–16.60)	0.59
Carbapenem administration	5 (71.4)	1 (5.3)	45.00 (3.35–603.99)	0.002

*CI* confidence interval; *ICU* intensive care unit; *IQR* interquartile range; *OR* odds ratio; *SD* standard deviation; -, not measured; *IQR* interquartile range; *ICU* intensive care unit.

### Intervention

After the intervention on 21^st^ October 2009, the cases decreased gradually and no further evidence of the outbreak strain was detected in December 2009 or January 2010. Follow-up ICU environmental surface cultures were processed monthly for 6 months after February 2010 and none grew MDR-Ab.

## Discussion

Although MDR-Ab is emerging more frequently in Chinese hospitals, [28,29] localized nosocomial outbreaks are rarely reported in China. The present study described a nosocomial bronchofiberscope-associated outbreak of *A. baumannii*. The significant association between bronchofiberscope use and MDR-AB incidence in this case–control study (Table 2) and the temporal association between bronchofiberscope use and MDR-Ab culture positivity (Figure 2) supported the conclusion. We also found MDR-Ab bronchofiberscope contamination and identified several potential administrative and technical problems with the in-ICU bronchofiberscope reprocessing practice. Nevertheless, univariate analysis revealed other significant risk factors, and the outbreak strain was not isolated from the bronchoscope only. Thus, the bronchofiberscope is among several rather than the only important factor that contributed to the outbreak.

In this study, genotype A MDR-Ab was defined as the outbreak strain. During the epidemic period, seven of 12 patients were infected or colonized with the outbreak strain, which was also isolated from multiple environmental surfaces within the ICU. Only one patient acquired the outbreak strain without direct bronchofiberscope exposure. Two patients were infected or colonized with MDR-Ab after intervention on 21^st^ October, one of whom had undergone bronchofiberscopy; however, these 2 MDR-Ab isolates were not identified as the outbreak strain, so the intervention definitely controlled the outbreak.

A similar large outbreak due to clonal MDR-Ab transmission has been reported, and widespread environmental contamination was perhaps promoted by the aerosolization of organisms during the pulsatile lavage debridement of infected wounds [10]. Our finding of an association between bronchofiberscopy and the MDR-Ab outbreak also highlights the importance of appropriate infection control measures when invasive medical procedures are performed. Since the environmental sample collection started before the infection control intervention measures were implemented, the cultures taken from the environment yielded high MDR-Ab rates. Eighty-five percent of the MDR-Ab isolates from the environmental samples were identical to the outbreak strain, indicating serious contamination of the surrounding environmental surfaces [25].

Importantly, four isolates collected directly from the non-disinfected and disinfected bronchofiberscope were also identified as being the outbreak strain, suggesting that serious failure of the bronchofiberscope reprocessing procedure and that the outbreak strain of MDR-Ab might have been transmitted through direct contact with the bronchofiberscope. Alternatively, these organisms could have been introduced into the environment by the index case or possibly by an unidentified patient and then transmitted through healthcare workers’ hands during other medical procedures; however, we did not identify the index case who “imported” the outbreak strain into the ICU, and no similar case was reported in other wards of the hospital.

In our investigation, most of the environmental MDR-Ab were isolated from the healthcare-associated environmental surfaces including the bed sheets, bedrails, dispensing table, nurses’ desk, and outer surface of the invigilator. There were no positive cultures collected from the healthcare workers’ hands or nasal cavities (data not shown), a finding that might be associated with high hand hygiene compliance rates during the investigation since all of the healthcare workers were concerned about the probable correlation between personnel contact and this MDR-Ab outbreak.

In addition to bronchofiberscopy treatment, univariate analysis of the case–control study showed that septic shock and renal disease were more common in the cases than in the controls. Similar results were also found that the underlying patient illness severity was a significant factor contributing to the acquisition of carbapenem-resistant *A. baumannii* in the ICU [30]. Moreover, length of ICU stay and the receipt of carbapenem were also risk factors [31,32]. Attempts were made to identify independent risk factors using multivariate logistic regression; however, the sample sizes were too small to allow for the drawing of reliable conclusions [5].

Bronchofiberscopy is used frequently within ICUs. Our findings emphasize that bronchofiberscopy must be performed with appropriate infection control measures. The present outbreak was not associated with bronchofiberscope defects or damage [20-22] but apparently was associated with its related cleaning and disinfection procedures. Therefore, strict bronchofiberscope reprocessing should be performed after each procedure and at the end of the day according to the published guidelines. It might be wise to increase the number of bronchofiberscopes available in each ICU to guarantee professional bronchofiberscope reprocessing within the hospital’s cleaning and disinfection department; however, this is usually limited for economic reasons, especially in less developed districts or countries. Therefore, assigning and training specific personnel to reprocess bronchofiberscopes in the ICU according to strict guidelines might also be a plausible solution. On the other hand, standard precautions must be implemented during bronchofiberscopy procedures, such as the use of personal protective equipment including fluid-resistant gowns, gloves, surgical masks, eye protection, and shoe and hair covers [32]. In addition, patients who receive bronchofiberscopy should be draped during treatment, and any potentially contaminated environmental surfaces should be thoroughly cleaned and disinfected after the procedure.

This outbreak was clinically significant due to the extensive antibiotic resistance of *A. baumannii* and the severity of the patient outcomes. Five of six cases who underwent bronchofiberscopy treatment developed MDR-Ab BSIs with severe clinical manifestations [13]. Half of the MDR-Ab carriers died in the ICU during the epidemic period, and MDR-Ab infection possibly contributed to four deaths. However, the significance of this case–control study is limited by its small sample size and wide 95% CIs.

The results of this case–control study demonstrated an association among factors but could not make a conclusion about causality. Further studies of similar outbreaks are needed to confirm these results. However, a strong association between bronchofiberscopy and MDR-Ab acquisition was confirmed by the epidemiologic and microbiologic analyses conducted during the outbreak.

## Competing interest

The authors declare that they have no competing interests.

## Authors’ contributions

The work was conducted together by Dep. Hospital Infection Control & Research, Institute for Disease Control & Prevention of Chinese People's Liberation Army and 309th Hospital of Chinese People's Liberation Army. LH conceived of the study and revised and approved the manuscript. YX conducted the microbiological study and drafted the manuscript. CL contributed to the study design and coordination and helped draft the manuscript. YC contributed to the data collection, analysis, and interpretation. FW participated in the environmental sampling process. BY contributed to the study design. JZ and GH contributed to the clinical document collection and data analysis. GJ carried out the molecular typing. XH and HL participated in the endoscopy sampling process. XD revised the manuscript to include important intellectual content. ZW contributed to the study design and performed the statistical analysis. HL contributed to the sampling and isolation of bacteria in the ICU. All authors read and approved the final manuscript.

## Pre-publication history

The pre-publication history for this paper can be accessed here:

http://www.biomedcentral.com/1471-2334/12/335/prepub
